# Innate immune responses to lysosomal nucleic acid stress

**DOI:** 10.1093/jb/mvaf011

**Published:** 2025-03-04

**Authors:** Kensuke Miyake, Takuma Shibata, Ryota Sato, Ryutaro Fukui

**Affiliations:** Division of Innate Immunity, The Institute of Medical Science, The University of Tokyo, 4-6-1 Shirokanedai, Minatoku, Tokyo 108-8639, Japan; Division of Innate Immunity, The Institute of Medical Science, The University of Tokyo, 4-6-1 Shirokanedai, Minatoku, Tokyo 108-8639, Japan; Division of Innate Immunity, The Institute of Medical Science, The University of Tokyo, 4-6-1 Shirokanedai, Minatoku, Tokyo 108-8639, Japan; Division of Innate Immunity, The Institute of Medical Science, The University of Tokyo, 4-6-1 Shirokanedai, Minatoku, Tokyo 108-8639, Japan

**Keywords:** DNase, nucleoside, RNase, transporter

## Abstract

Nucleic acids (NAs) are recognized by endosomal Toll-like receptors (TLRs) and cytoplasmic sensors in innate immune cells. NAs accumulate within lysosomes due to either excessive NA influx or defective lysosomal degradation. The resultant storage of NAs and/or NA metabolites in the lysosome, referred to here as lysosomal NA stress, elicits a spectrum of responses, ranging from inflammation to tissue repair, through NA sensor activation. Although these responses contribute to host defence against infection, they may also drive diseases. For instance, loss of function of the lysosomal nucleoside transporter SLC29A3 drives lysosomal nucleoside stress, which activates TLR8 in macrophages to cause histiocytic diseases collectively called SLC29A3 disorders. Similarly, DNase II deficiency promotes lysosomal DNA stress, leading to activation of cytoplasmic double-stranded DNA sensors, such as cGAS-STING and AIM2, and thereby autoinflammatory and autoimmune diseases. Thus, lysosomal NA stress is viewed as a pivotal environmental signal that initiates innate immune responses.

## Graphical Abstract

**Graphical Abstract:**
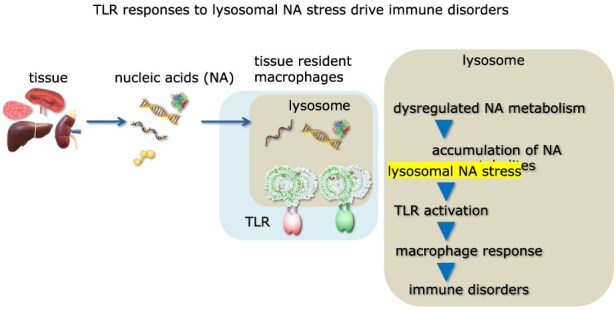

Extracellular nucleic acids (NAs) are internalized by macrophages and degraded within lysosomes. Tissue damage increases NA influx into lysosomes, whereas defects in lysosomal degradation leads to NA accumulation, which hereafter is referred to as ‘lysosomal NA stress’. In innate immune cells, including macrophages, NA sensors are located in both lysosomes and the cytoplasm. Although these sensors typically recognize pathogen-derived NAs, they can also respond to self-derived NAs that accumulate during tissue damage. Consequently, NA sensors alert innate immune cells to both infection and tissue damage. This stress response, mediated by NA sensors, can drive diseases such as autoinflammatory, autoimmune and histiocytic diseases through macrophage-mediated inflammation, lymphocyte-mediated inflammation and macrophage proliferation, respectively *(**1**,*  *2**)*. The specific type of accumulated NAs and/or NA metabolites influences the immune response and disease outcome. In this review, we summarize current understanding of how lysosomal NA stress engages innate immune responses and contributes to disease pathogenesis.

## Toll-Like Receptors

Toll-like receptors (TLRs) directly bind to pathogen-derived components. For instance, bacterial lipopolysaccharides and lipopeptides are detected by TLR4/MD-2 and TLR2 heterodimers such as TLR2/TLR1 and TLR2/TLR6. These cell surface TLRs are regarded as pathogen sensors in the mammalian innate immune system due to the pathogen-specific nature of lipopolysaccharides and lipopeptides. In contrast, NAs are recognized by TLRs such as TLR3, TLR7, TLR8, TLR9 and TLR13. Among these, TLR9 responds more strongly to bacterial double-stranded DNAs (dsDNAs) than self-derived DNAs based on sequences and methylation patterns *(**3**)*. However, TLR9 and TLR7 have been implicated in various autoimmune diseases such as systemic lupus erythematosus (SLE) and psoriasis *(**4**,*  *5**)*, suggesting that endosomal TLRs can detect self-derived NAs as indicators of cellular stress and tissue damage. Moreover, TLR3 activation has been shown to promote tissue repair following tissue injury *(**6**)*. Collectively, these findings imply that endosomal TLRs function as both pathogen and stress sensors, mediating responses that span from defence response against infections to tissue repair. Notably, TLR responses to self-derived NAs are markedly induced under lysosomal NA stress.

## Cytoplasmic NA Sensors

dsDNAs in the cytoplasm are sensed by the cyclic GMP-AMP synthase (cGAS)-stimulator of interferon genes (STING) pathway and the absent in melanoma 2 (AIM2) sensor *(**7*–*9**)*. Activation of cGAS-STING drives the production of proinflammatory cytokines and type I interferons (IFNs), whereas AIM2 initiates inflammasome assembly. STING is primarily localized in the endoplasmic reticulum (ER) but traffics to the Golgi apparatus to activate downstream signalling. In parallel, cytoplasmic dsRNA is sensed by retinoic acid-inducible gene I (RIG-I) and melanoma differentiation-associated protein 5 (MDA5) *(**10**)*, which engage mitochondrial antiviral-signalling protein (MAVS) to induce production of proinflammatory cytokines and type I IFNs.

## Lysosomal NA Metabolism and Its Impact on NA Sensors

**Fig. 1:**
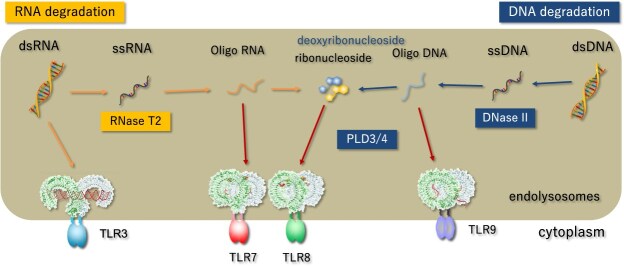
**NA degradation and NA sensing in the lysosome.** Extracellular NAs are internalized by macrophages and degraded by nucleases, including RNase T2, DNase II, PLD3 and PLD4. NA-sensing TLRs reside within lysosomes, where they detect NAs and/or their degradation products. Some NA degradation is required for NA sensing by TLR7, TLR8 and TLR9, whereas other NA degradation negatively regulates NA sensing by digesting TLR ligands.

Extracellular and cytoplasmic NAs are transported into lysosomes, where DNases and RNases degrade them to nucleosides. These nucleosides are subsequently exported to the cytoplasm through the nucleoside transporter SLC29A3. Within lysosomes, TLR3, TLR7/TLR8 and TLR9 recognize NAs and their degradation products. Lysosomal NA metabolism exerts both positive and negative effects on TLR responses by generating or degrading TLR ligands, respectively. For example, RNase T2 negatively regulates TLR3 by accelerating dsRNA degradation, but it is also required to generate TLR7/8 ligands *(**11*–*13**)*. DNase II produces ssDNA that activates TLR9 *(**14**)*, yet it concurrently limits dsDNA sensing by cGAS-STING *(**15**)*. Phospholipase D3 (PLD3) and PLD4 function as exonucleases that degrade NA, thereby inhibiting TLR7, TLR9 and cGAS *(**16**,*  *17**)*, although they positively regulate TLR7 signalling by generating its ligand *(**18**)*. Alterations in lysosomal NA metabolism can lead to the accumulation of NAs and/or their degradation products, thereby triggering TLR activation (Fig. 1).

**Fig. 2:**
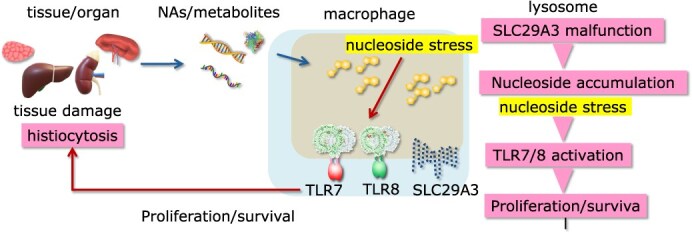
**Nucleoside stress drives histiocytosis by activating TLR7 and 8.** Loss of function variations in the lysosomal nucleoside transporter SLC29A3 induce lysosomal nucleoside stress in macrophages, thereby activating TLR7 and TLR8, and driving histiocytosis characterized by macrophage accumulation in multiple organs.

## Lysosomal Nucleoside Stress Activates TLR7 and TLR8

The nucleoside transporter SLC29A3, a lysosomal multiple-transmembrane protein highly expressed in macrophages, is localized to endosomal compartments and operates optimally at pH 5.5. Loss-of-function variations in the SLC29A3 gene underlie a group of histiocytic diseases collectively referred to as SLC29A3 disorders, characterized by histiocytosis, hepatosplenomegaly, skin pigmentation, hypertrichosis and type I diabetes (Fig. 2) *(**2**,*  *19**)*. Mice lacking *Slc29a3* exhibit similar pathological features, including marked accumulation of macrophages in the spleen *(**20**)*. Although nucleosides accumulate within macrophage lysosomes, the mechanism by which they drive histiocytosis remained unclear until recently. New evidence indicates that nucleoside accumulation activates TLR7 and TLR8, promoting histiocytosis in *Slc29a3*^−/−^ mice and in SLC29A3 disorder patients, respectively *(**21**,*  *22**)*. Although mouse TLR8 is expressed in innate immune cells, it does not respond to TLR8 agonists *(**23**)*. Therefore, macrophage accumulation in *Slc29a3*^−/−^ mice is completely abolished by the lack of TLR7, suggesting that TLR7-mediated signalling pathways are essential for histiocytosis development in mice. Flowcytometry shows that TLR7-dependent signals increase the numbers of monocyte progenitors, induce proliferation of immature monocytes and lead to an accumulation of Ly6C^low^ mature macrophages in the spleen. These findings indicate that TLR7 in *Slc29a3*^−/−^ mice initiates emergency myelopoiesis *(**24**,*  *25**)*, a process typically observed during infections. Transcriptome analyses of splenic macrophages reveal TLR7-dependent proliferation and attenuation of inflammatory responses, indicating that lysosomal nucleoside stress does not trigger inflammatory responses. In humans, both TLR7 and TLR8 function in macrophages *(**21**)*. The TLR8 antagonist, but not the TLR7 antagonist, completely inhibited enhanced survival and proliferation *in vitro* of peripheral blood monocytes harbouring a pathogenic G208R *SLC29A3* variation *(**21**)*. This observation suggests that TLR8, not TLR7, drives survival and proliferation of monocytes in human SLC29A3 disorders. The role of TLR7 in SLC29A3 disorders remains unknown.

## Lysosomal RNA Stress Activates TLR13 in Mice

Murine innate immune cells express RNases belonging to type A and T2 families. For instance, bone marrow (BM)-derived macrophages express RNase 4 and RNase T2, while BM-derived plasmacytoid dendritic cells (BM-pDCs) express RNase 6 and RNase T2 *(**11**)*. In BM-conventional DCs (BM-cDCs), RNase T2 expression is notably high. Among these enzymes, RNase T2 has been shown to modulate TLR responses. RNase T2, the only member of the T2 RNase family, consisting of 256 amino acids, is ubiquitously expressed, and localized to lysosomes where it degrades RNA under acidic conditions. Loss-of-function variations in the *RNASET2* gene cause cystic leukoencephalopathy without megalencephaly in humans *(**26**)*, a disease characterized by bilateral anterior temporal subcortical cysts, multifocal white matter lesions, psychomotor impairment, spasticity and epilepsy *(**27**)*. Consistently, *Rnaset2*^−/−^ mice exhibit type I IFN-dependent neuroinflammation *(**28**)*.

**Fig. 3:**
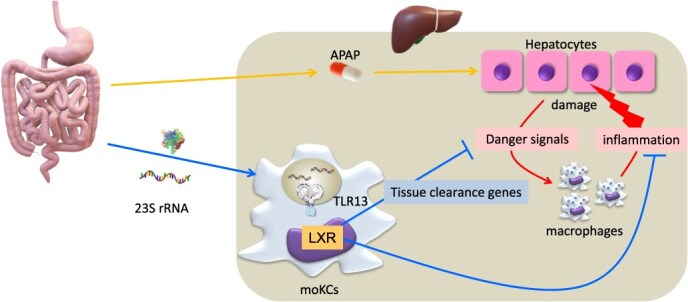
**RNA stress in Kupffer cells drives tissue repair response through TLR13 activation.** RNase T2 deficiency results in lysosomal RNA stress, characterized by an accumulation of species that include the TLR13 ligand, bacterial 23S ribosomal RNA derived from the gut microbiota. TLR13 signalling within moKCs leads to the activation of the transcription factor LXR, which upregulates tissue clearance genes and attenuates inflammatory pathways. Consequently, the enhanced clearance of danger signals and diminished inflammatory response render hepatocytes more resistant to acetaminophen (APAP)-induced liver injury.

RNase T2 negatively regulates TLR3 responses by degrading dsRNA *(**11**)*, but positively regulates mouse TLR7 and human TLR8 by generating the ligands they recognize *(**11*–*13**)*. In *Rnaset2*^−/−^ mice, splenomegaly and hepatomegaly develop *(**29**)*, while *Slc29a3*^−/−^ mice show only splenomegaly. TLR13 deficiency abolishes hepatosplenomegaly in *Rnaset2*^−/−^ mice, indicating that TLR13 drives macrophage expansion. TLR13, which recognizes ssRNA from bacterial 23S ribosomal RNA (rRNA) *(**30**,*  *31**)*, is expressed in macrophages, cDCs and neutrophils in mice, but absent in humans. Antibiotics treatment rescues hepatosplenomegaly in *Rnaset2*^−/−^ mice, suggesting that bacterial rRNAs from the gut reach the liver via the portal vein.

**Fig. 4:**
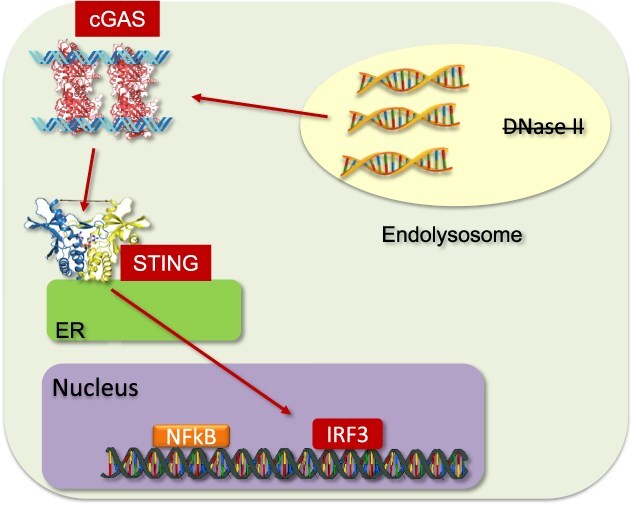
**Lysosomal DNA stress activates cytoplasmic dsDNA sensors.** DNase II deficiency induces lysosomal DNA stress, permitting accumulated DNA to translocate into the cytoplasm and engage dsDNA sensors such as cGAS-STING and AIM2. This activation drives autoinflammatory and autoimmune responses.

**Fig. 5:**
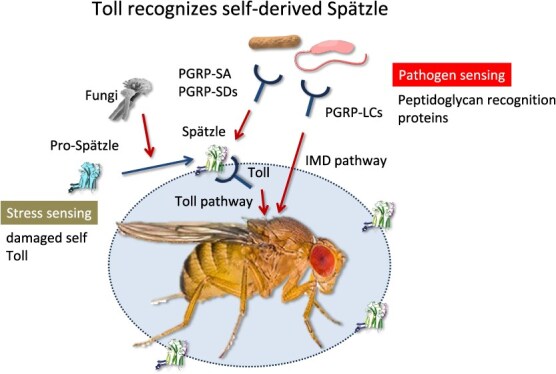
**Drosophila Toll is a stress sensor.** Drosophila Toll is activated during infections. Fungal infections cleave pro-Spätzle into its active form. The resultant Spätzle-Toll interaction induces antimicrobial peptide production, underscoring the role of Toll as a stress sensor. Meanwhile, PGRPs function as dedicated pathogen sensors by binding bacterial peptidoglycans and activating either the Toll or IMD pathway. Thus, Drosophila employs both pathogen- and stress-sensing mechanisms to combat infections.

Further investigation shows that TLR13 promotes emergency myelopoiesis, producing macrophages that infiltrate the spleen and liver in *Rnaset2*^−/−^ mice. Within the spleen, TLR13 continuously drives macrophage proliferation, resulting in an accumulation of IL-10-producing monocytes. In the liver, TLR13 causes accumulation of monocyte-derived Kupffer cells (moKCs) *(**32**)*. These moKCs exhibit activation of the transcription factors LXR and MafB, and contribute to tissue repair, rendering the liver resistant to drug-induced liver injury caused by acetaminophen. Notably, acetaminophen triggers hepatocyte damage, release of danger signals from damaged hepatocytes, macrophages activation and inflammation-induced hepatocyte damage, leading to liver failure *(**33**)*. In *Rnaset2*^−/−^ mice, although initial hepatocyte damage occurs, it is not followed by an inflammatory response because TLR-dependent cytokine production is impaired and the gene involved in tissue clearance, e.g. CD5L/apoptosis inhibitor of macrophage (AIM), MerTK and AXL, are upregulated. Administration of CD5L/AIM protein mitigates acetaminophen-induced liver injury by promoting clearance of dead cells and danger signals *(**34**,*  *35**)*. Similarly, MerTK and AXL facilitate the removal of dying cells *(**36**)*. Taken together, these findings suggest that TLR13 signalling in response to bacterial rRNA initiates tissue clearance and repair responses in moKCs (Fig. 3).

## Lysosomal DNA Stress Activates Endosomal and Cytoplasmic DNA Sensors

DNase II is a ubiquitously expressed lysosomal DNase. Its deficiency causes lethal anaemia due to constitutive type I IFN production *(**37**,*  *38**)*. Although DNA accumulate in lysosomes, TLR9 does not mediate this lethal anaemia *(**39**)*. Because TLR9 activation depends on DNA digestion by DNase II *(**14**)*, undigested DNA cannot properly activate TLR9. Instead, lysosomal DNA appears to reach the cytoplasm, activating the cytoplasmic dsDNA sensor cGAS (Fig. 4), which triggers type I IFN production *(**15**)*. In the absence of the type I IFN receptor, *Dnase2a*^−/−^ mice are born normally but develop TNF-α-dependent arthritis *(**40**)*. In addition to cGAS, AIM2 also contributes to arthritis development, while endosomal TLRs drive autoantibody production *(**41**)*. The precise mechanism by which lysosomal dsDNA translocates to the cytoplasm remains to be clarified. Clinically, loss-of-function variants in *DNASE2* lead to severe neonatal anaemia, glomerulonephritis, liver fibrosis, arthropathy and a transcriptomic signature of elevated type I IFN signalling *(**42**)*. These manifestations are consistent with the phenotypes in *Dnase2a*^−/−^ mice.

In the circulation, DNase 1 and DNase 1-like 3 (DNase 1 l3) are enzymatically active *(**43**)*. DNase 1 is expressed in nonhematopoietic tissue such as the salivary gland, lacrimal gland and kidney *(**44**)*, whereas DNase 1 l3 is expressed in cDCs and macrophages. The former preferentially degrades protein-free DNA, whereas the latter targets chromatin in microparticles *(**45**)*. These DNases degrade DNAs in neutrophil extracellular trap (NET), the complex of chromatin and proteins released from dying neutrophils *(**43**)*. Impaired degradation of NET predisposes to lupus nephritis *(**46**)*. Consistently, loss-of-function variations in *DNASE1* or *DNASE1L3* predispose to SLE *(**47**,*  *48**)*, suggesting that undigested DNAs activate B cells, macrophages and DCs in *Dnase1l3*^−/−^ mice, leading to lupus nephritis *(**49**)*. Unexpectedly, TLR7, in addition to TLR9, mediates autoimmune responses in Dnase1l3−/− mice. TLR7 is likely to respond to DNA-derived deoxyguanosine with endogenous oligo RNA in *Dnase1l3*^−/−^ mice *(**21**)*.

PLD3 and PLD4 are lysosomal type II transmembrane proteins that have two independent functions, degradation of nucleic acids and synthesis of the lysosomal phospholipid S,S-Bismonoacyl glycerophophate (BMP) *(**16**,*  *50**)*. The absence of PLD3 and PLD4 activates multiple NA sensors—including TLR9, TLR7 and the cGAS-STING pathway—resulting in lethal hemophagocytic lymphohistiocytosis characterized by liver damage and elevated IFN-γ production. These inflammatory phenotypes likely stem from lysosomal NA stress and/or lysosomal dysfunction due to reduced BMP levels. Variations in the *PLD3* and *PLD4* genes are linked with Alzheimer’s disease and autoimmune diseases, such as systemic sclerosis and rheumatoid arthritis *(**51*–*53**)*, respectively. Activation of NA sensors may thus contribute to these PLD3/4-associated disorders.

Taken together, lysosomal DNA stress can promote autoinflammatory and autoimmune diseases via TLR9, cGAS-STING and AIM2 activation.

## Nucleic Acid Stress Response during Infections

In the case of Herpes Simplex Virus (HSV) infection, at least three distinct pathways mediate detection of the virus. First, HSV-derived DNA activates the cGAS-STING pathway to induce type I IFN production. In this situation, the cGAS serves as a pathogen sensor. Second, HSV infection induces mitochondrial stress, leading to the release of mitochondrial dsDNA that also activates cGAS-STING *(**54**)*. In this setting, cGAS-STING serves as a stress sensor. cGAS-STING does not discriminate between viral and mitochondrial DNAs. Third, HSV infection is sensed by endosomal TLR3, which detects HSV-derived and/or self-derived dsRNA for type I IFN production. Remarkably, this TLR3-dependent pathway is critical in neurons; loss-of-function variants in *TLR3* predispose to HSV encephalitis *(**55**)*. In Neuronal cells, TLR3 appears more crucial than cGAS-STING for HSV control *(**56**)*. These findings demonstrate that infections elicit innate immune responses against both pathogen-derived and self-derived NAs.

## Concluding Remarks

Drosophila Toll is the prototype for mammalian TLRs and indispensable for antifungal immunity *(**57**)*. However, its ligand is the self-derived molecule Spätzle, which undergoes proteolytic cleavage upon fungal infection *(**58**)*. Thus, Drosophila Toll functions primarily as a stress sensor rather than a direct pathogen sensor. Because Toll is essential for defence against fungal infection, Drosophila likely lacks a dedicated receptor for fungal components. On the other hand, for defence against bacterial infections, peptidoglycan recognition proteins (PGRPs) detect bacterial peptidoglycans, activating the Toll and IMD pathways *(**59**)*. Drosophila, akin to mammals, harness both pathogen- and stress-sensing machinery to combat infections (Fig. 5). Given that stress sensing is evolutionarily conserved from Drosophila to mammals, detecting alteration in self-derived molecules is as vital as recognizing pathogen-associated molecules to combat infections. NA is one of the self-derived molecules that are watched by innate immune sensors.
